# Bayesian Re-analysis of Two-Year Outcomes in the Platelet-Rich Plasma in Achilles Tendon Healing 2 (PATH-2) Trial: Platelet-Rich Plasma for Achilles Tendon Rupture

**DOI:** 10.7759/cureus.102519

**Published:** 2026-01-28

**Authors:** Justin Le, Milan Patel, Andrew Atschinow, Anish Rana, Gilbert Siu

**Affiliations:** 1 Physical Medicine and Rehabilitation, Rowan-Virtua School of Osteopathic Medicine, Stratford, USA; 2 Physical Medicine and Rehabilitation, Touro College of Osteopathic Medicine, Middletown, USA; 3 Physical Medicine and Rehabilitation, University of Rochester School of Medicine and Dentistry, Rochester, USA

**Keywords:** acute achilles tendon rupture, ankle and foot, functional score, outcome analysis, platelet-rich plasma (prp)

## Abstract

The platelet-rich plasma in achilles tendon healing 2 (PATH-2) trial is the largest randomized study of platelet-rich plasma (PRP) in acute Achilles tendon rupture. While the original frequentist analysis showed no significant benefit, such methods cannot quantify the probability of clinically meaningful improvement. We conducted a Bayesian re-analysis of PATH-2 data (n = 177) with Achilles tendon total rupture score (ATRS) and patient-specific functional scale (PSFS) at 24 months as outcomes. Minimally clinically important differences (MCIDs) were defined as 8 ATRS points and 2.3 PSFS points. Across flat, neutral, optimistic, and pessimistic priors, posterior probabilities of PRP providing benefit ≥MCID were <0.001% for ATRS and ~5×10^-13^ for PSFS. Probabilities of any benefit were 39.6% and 47.0%, respectively, with posterior mean differences close to zero. Results were robust across priors. This analysis demonstrates that PRP is unlikely to yield clinically meaningful functional improvement after Achilles tendon rupture.

## Introduction

Acute Achilles tendon rupture is a common and disabling injury, particularly among physically active individuals, with prolonged recovery and risk of incomplete functional restoration [1,2]. Recent strategies in biological augmentation have been investigated to improve tendon healing and long-term outcomes. Among these, platelet-rich plasma (PRP) has gained significant popularity in musculoskeletal medicine due to its concentration of platelets and growth factors with purported regenerative effects [3,4].

PRP has been marketed heavily and is often requested by athletes and patients willing to pay out-of-pocket [5,6]. Achilles tendon rupture, with its prevalence in younger, active individuals, has been a natural target for biologic enthusiasm [7]. However, despite theoretical appeal, repeated studies have shown no benefit [7-12]. Reported outcomes depend on injury type, anatomical site of injection, and PRP formulation. Inconsistent reporting may additionally complicate the interpretation of its benefit. The platelet-rich plasma in Achilles tendon healing (PATH-2) randomized controlled trial (RCT), the largest study of PRP in acute Achilles tendon rupture, initially reported no statistically significant difference in functional recovery between PRP and placebo at short-term follow-up of 24 weeks [8]. Subsequently, a 24-month follow-up analysis was published, which confirmed the absence of clinically relevant benefit, showing similar Achilles tendon total rupture score (ATRS) and patient-specific functional scale (PSFS) outcomes between groups [9]. Despite this, uncertainty remains around whether PRP may confer even a small but clinically meaningful benefit.

While these frequentist analyses provide incredibly valuable evidence, they remain limited by reliance on p-values and confidence intervals, which cannot quantify the probability of treatment benefit or the likelihood of achieving clinically meaningful improvement, outcomes most relevant for clinicians and patients considering PRP. Bayesian methods, in contrast, allow integration of prior knowledge and yield probability distributions for treatment effects, therefore quantifying the probability of a treatment being clinically meaningful and providing any benefit exceeding the minimal clinically important difference (MCID) [13]. Specifically, Bayesian modeling can demonstrate whether or not the probability of PRP yielding even a modest functional gain is low, as well as the likelihood of surpassing validated MCIDs.

Objective

To perform a Bayesian re-analysis of the PATH-2 trial 24-month outcomes using neutral and informative prior assumptions to quantify the probability that PRP for Achilles tendon rupture provides (1) any functional benefit and (2) clinically meaningful improvement exceeding pre-specified MCIDs.

## Materials and methods

Study design and data source

We conducted a Bayesian re-analysis of the PATH-2 trial, which investigated the effect of PRP on functional recovery following Achilles tendon rupture. In the trial, leukocyte-rich, five-fold concentrated PRP was prepared from up to 55 mL of autologous blood using a standardized centrifugation system and administered as a single injection into the Achilles tendon rupture gap within 12 days of injury. Only aggregate summary data were used; no patient-level data were available [9]. Group means and standard errors were extracted directly from the trial report for both primary and secondary outcomes (Table 1).

**Table 1:** PATH-2 trial data used for the Bayesian model Mean differences are reported as the PRP group minus the control group. CI, Confidence Interval; SE, standard error Copyright/License: The data are adopted from Keene et al. [9], which is an open-access article distributed under the terms of the Creative Commons Attribution (CC BY 4.0) licence.

Study	Outcome	Timepoint	Mean Difference (MD)	Lower 95% CI	Upper 95% CI	SE	N (analyzed)
PATH-2 follow-up (BJJ 2022) [9]	ATRS (0-100)	24 months	-0.752	-5.523	4.02	2.435	177 (85 PRP, 92 control)
	PSFS (0-10)	24 months	-0.023	-0.618	0.573	0.304	177 (85 PRP, 92 control)

Outcomes and MCIDs

The pre-specified primary outcome was the ATRS, a 0-100 scale with higher scores indicating better function [14]. The MCID was defined as eight points based on prior validation studies of the ATRS [15]. The secondary outcome was the PSFS, a 0-10 scale assessing patient-selected functional tasks, with an MCID defined as 2.3 points, consistent with musculoskeletal rehabilitation literature [16,17].

Bayesian model specification

The treatment effect was defined as the mean difference in outcome scores between the PRP and control groups. Analyses were based on group means and standard errors from the PATH-2 trial. Using a Bayesian normal-normal model, we assumed a normal likelihood for the observed treatment effect: y ~ Normal(θ, σ²), where y is the observed mean difference, σ² is the reported variance, and θ represents the true treatment effect.

For the ATRS (0-100 scale; MCID = 8 points), we specified four prior distributions on θ: a flat prior Normal(0, 1000) assuming no preconceived expectations, a neutral prior Normal(0, 4) assuming no benefit or harm in either direction, an optimistic prior Normal(2, 4) leaning to small benefit, and a pessimistic prior Normal(-2, 4) leaning to worsening with treatment. The variance of 4 (SD = 2) was chosen to reflect modest prior uncertainty centered around no effect, with most prior mass falling within ±4 points, approximately half of the MCID.

For the PSFS (0-10 scale; MCID = 2.3 points), we specified analogous priors: a flat prior Normal(0, 1000), a neutral prior Normal(0, 2), an optimistic prior Normal(2.3, 2), and a pessimistic prior Normal(-2.3, 2). Here, the variance of 2 (SD ≈ 1.4) was selected so that the priors place most weight within ±3 points of the mean, corresponding to roughly one MCID on the PSFS scale. Posterior distributions were obtained analytically through conjugate Bayesian updating. From each posterior, we estimated the posterior mean, 95% credible interval (CrI), and posterior probabilities for any benefit (θ > 0) and clinically meaningful benefit (θ ≥ MCID).

Sensitivity analysis

To assess robustness, we performed sensitivity analyses across all four prior specifications for each outcome. This allowed evaluation of the extent to which conclusions were influenced by optimistic, pessimistic, or neutral prior beliefs, as well as by an essentially non-informative flat prior.

Statistical software

All analyses were performed in R (version 4.5.1; R Foundation for Statistical Computing, Vienna, Austria). Bayesian posterior estimates were obtained using a normal-normal conjugate model, implemented with base R functions (qnorm, pnorm, rnorm). For each outcome, posterior means, standard deviations, and 95% credible intervals were computed, and posterior probabilities were estimated for any benefit and for benefit exceeding the prespecified MCID thresholds. Flat, neutral, optimistic, and pessimistic priors were specified as normal distributions with predefined means and variances. Data wrangling was performed using the dplyr package (v1.1.4), and visualizations (forest plots, posterior density plots) were generated using ggplot2 (v3.5.2).

IRB statement

All analyses used published aggregate PATH-2 data, and no direct patient data was accessed. IRB approval was not required prior to conducting this study.

## Results

For the ATRS at 24 months, the neutral prior yielded a posterior mean difference of -0.549 (95% CrI -4.623 to 3.528) between PRP and control groups. The posterior probability of any benefit (θ > 0) was 39.6%, while the probability of achieving a clinically meaningful improvement (≥8 ATRS points) was <0.001%. Findings were consistent across prior assumptions, with posterior mean differences ranging from -1.089 to -0.008 and uniformly negligible probabilities of benefit ≥ MCID (Table 2).

**Table 2:** Achilles tendon total rupture score under varying prior assumptions in a Bayesian normal-normal model SD, standard deviation; CrI, credible interval; Pr, posterior probability; ATRS, Achilles tendon total rupture score

ATRS	Prior (mean, SD)	Threshold	Posterior mean difference	95% CrI	Pr (any benefit)	Pr (benefit ≥ threshold)
Flat	0, 1000	8 ATRS	-0.752	-5.525 to +4.021	0.378	0.000163
Neutral	0, 4	8 ATRS	-0.549	-4.623 to +3.528	0.396	0.000198
Optimistic	2, 4	8 ATRS	-0.008	-4.085 to +4.069	0.498	0.000059
Pessimistic	-2, 4	8 ATRS	-1.089	-5.166 to +2.987	0.3	0.000062

For the PSFS at 24 months, the neutral prior produced a posterior mean difference of -0.022 (95% CrI: -0.612 to 0.567). The probability of any benefit was 47.0%, and the probability of benefit ≥ MCID (≥2.3 points) was approximately 5×10^-13^. Results under flat, optimistic, and pessimistic priors showed similar distributions, with all posterior probabilities of clinically meaningful benefit effectively zero (Table 3).

**Table 3:** Patient-specific functional scores under varying prior assumptions in a Bayesian normal-normal model SD, standard deviation; CrI, credible interval; Pr, posterior probability; PSFS, patient-specific functional scale

PSFS	Prior (mean, SD)	Threshold	Posterior mean difference	95% CrI	Pr (any benefit)	Pr (benefit ≥ threshold)
Flat	0, 1000	2.3 PSFS	-0.023	-0.619 to +0.573	0.47	1.08 x 10^-12^
Neutral	0, 2	2.3 PSFS	-0.022	-0.612 to +0.567	0.47	5.44 x 10^-13^
Optimistic	2.3, 2	2.3 PSFS	0.029	-0.560 to +0.619	0.539	2.10 x 10^-12^
Pessimistic	-2.3, 2	2.3 PSFS	-0.074	-0.663 to +0.515	0.402	1.44 x 10^-13^

**Figure 1:**
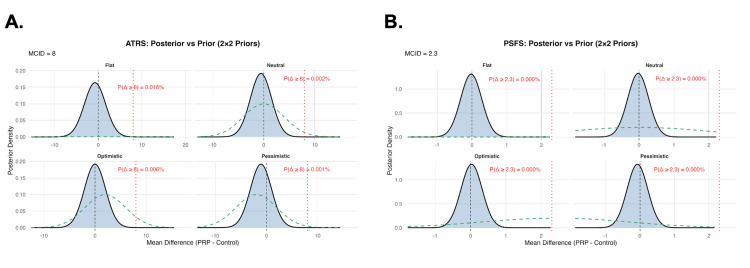
Posterior and prior distributions from a Bayesian normal-normal model under four prior assumptions (A) Posterior distributions of the mean difference in ATRS between PRP and control groups under flat, neutral, optimistic, and pessimistic priors. Shaded curves represent posterior densities, dashed green curves represent priors, the vertical dashed line indicates no effect, and the vertical dotted red line denotes the MCID, set at 8 points. Posterior probabilities of clinically meaningful benefit (Δ ≥ 8) are shown. (B) Posterior distributions of the mean difference in PSFS scores under the same prior assumptions. Visual elements are as in Panel A, with the MCID set at 2.3 points. ATRS, Achilles tendon total rupture score; PSFS, patient-specific functional scale

Posterior density plots illustrate the consistency of these findings across prior assumptions, with the distributions tightly centered near zero and far below the MCID thresholds for both ATRS and PSFS (Figure 1). Overall, across both functional outcomes, the Bayesian re-analysis demonstrated a high likelihood that PRP confers no clinically meaningful advantage over control at 24 months following Achilles tendon rupture.

## Discussion

This Bayesian re-analysis of the PATH-2 trial reinforces the conclusion that PRP is highly unlikely to offer a clinically meaningful benefit in the management of acute Achilles tendon rupture. The original trial, a multicenter randomized placebo-controlled study of more than 200 patients, demonstrated no advantage of PRP over placebo in patient-reported function or tendon-specific outcomes at 24 weeks and two years of follow-up [5,6]. Our analysis does not contradict these findings and instead adds a new perspective by quantifying the probability of benefit.

Traditional frequentist analyses communicate results through p-values and confidence intervals, which are often interpreted dichotomously as “positive” or “negative” [18-20]. A non-significant p-value, however, does not indicate the absence of effect, but rather, it only suggests that the trial was unable to reject the null hypothesis at a predefined threshold. For clinicians and patients, a more intuitive question is as follows: What is the probability that this intervention provides a clinically meaningful benefit? Bayesian methods help provide that answer more directly [13,21]. In this analysis, posterior distributions overwhelmingly excluded the possibility of meaningful benefit, regardless of whether optimistic, neutral, flat, or skeptical priors were assumed. The importance of this re-analysis lies not in overturning a negative trial, but in clarifying its implications within a field where biologics continue to diffuse into practice despite mixed evidence.

Other randomized trials support our findings and conclusions. Schepull et al. randomized patients undergoing operative repair to intraoperative autologous platelets or control and found no differences in tendon healing or mechanical strength [11]. Boesen et al. conducted a placebo-controlled trial of four PRP injections in nonsurgical ruptures and observed no between-group differences in ATRS, heel-rise, tendon elongation, or calf circumference through 12 months [12]. Keene et al. tested delayed PRP injection three weeks after surgical repair and similarly found no meaningful improvement over placebo [8,9]. Yasui et al. most recently confirmed the absence of benefit in a double-blind, ultrasound-guided postoperative trial with two-year follow-up [22].

Meta-analyses across tendon disorders reinforce these findings as well, showing consistent futility with no reproducible evidence of benefit. A Cochrane review concluded that there is insufficient evidence to support PRP for musculoskeletal soft tissue injuries, citing high heterogeneity and lack of consistent efficacy [23]. Ali Elsiddig Ahmed et al. similarly found no reproducible evidence of benefit across tendon disorders [24]. Reviews focused on Achilles rupture have drawn the same conclusion: across operative and nonoperative care, PRP has not improved functional outcomes [10].

While PATH-2 was conclusive using frequentist methods, Bayesian analysis adds clinical clarity. Instead of saying PRP “failed to reach statistical significance,” we can state that PRP has less than 1% probability of achieving an improvement greater than the MCID. Such phrasing may better resonate with clinicians, payers, and guideline committees tasked with deciding whether an intervention should be adopted or reimbursed. In addition, Bayesian methods are increasingly applied in medicine where interventions are costly or prematurely adopted [25]. Secondary Bayesian re-analyses in stroke and intracerebral hemorrhage have demonstrated how probability-based framing can contextualize null results [26]. Sports medicine and orthopedics, where biologics remain widely promoted, are particularly well-suited to benefit from such approaches.

Strengths of this study include reliance on the largest and most rigorously designed PRP trial in Achilles rupture, the use of multiple priors to test robustness, and the anchoring of findings to validated MCIDs. These enhance both statistical credibility and clinical interpretability. Limitations should also be noted. Without access to patient-level data, subgroup analyses were not possible, and generalizability to alternative PRP formulations or dosing regimens remains uncertain. As with any Bayesian analysis, priors influence posterior results, though the consistency across skeptical, neutral, and optimistic priors mitigates this concern. Finally, the PATH-2 trial evaluated a single PRP formulation, leukocyte-rich PRP at approximately fivefold platelet concentration administered as a single injection; while PRP represents a heterogeneous intervention space encompassing variable leukocyte content, platelet concentrations, activation methods, delivery techniques, and repeat-dosing strategies that were not examined. Variation across these parameters may meaningfully influence recovery.

Overall, future research on biologics could benefit from including Bayesian methods prospectively. Adaptive trial designs informed by posterior probabilities could allow for earlier termination of futile interventions, more efficient sample sizes, and clearer communication of results. Identifying potential responder subgroups, guided by biomarkers or imaging characteristics, may clarify whether niche populations could benefit from PRP or similar biologics. In the absence of such data, existing randomized trials, systematic reviews, and the present Bayesian re-analysis are broadly consistent in demonstrating no clear evidence of a clinically meaningful benefit of PRP for acute Achilles tendon rupture recovery.

## Conclusions

This re-analysis of the PATH-2 trial provides a probabilistic reinterpretation of treatment effects associated with PRP in acute Achilles tendon rupture. By incorporating multiple prior assumptions, the analysis demonstrates that across skeptical, neutral, and optimistic priors, the posterior probability of achieving a clinically meaningful improvement in function remains low. Bayesian probability framing complements the original frequentist findings by explicitly quantifying uncertainty around treatment benefit for the specific PRP formulation, dosage, and injection protocol evaluated in PATH-2. While these findings do not support a clinically meaningful effect in this context, they do not preclude the possibility that alternative PRP preparations, dosing strategies, or delivery methods could yield different outcomes. Future randomized trials may benefit from standardized biologic protocols and pre-specified Bayesian analyses to more efficiently characterize treatment effects and uncertainty.
